# Perceived environment and public open space use: a study with adults from Curitiba, Brazil

**DOI:** 10.1186/1479-5868-10-35

**Published:** 2013-03-15

**Authors:** Rogério César Fermino, Rodrigo Siqueira Reis, Pedro Curi Hallal, José Cazuza de Farias Júnior

**Affiliations:** 1 Pontifícia Universidade Católica do Paraná, School of Health and Biosciences, Curitiba, Brazil; 2 Universidade Federal do Paraná, Curitiba, Brazil; 3 Universidade Federal de Pelotas, Pelotas, Brazil; 4 Universidade Federal da Paraíba, João Pessoa, Brazil

**Keywords:** Built environment, Physical environment, Green areas, Public parks, Recreational facilities

## Abstract

**Background:**

The aim of this study was to investigate the association between the perceived environment and the use of public open spaces (POS).

**Methods:**

A cross-sectional study with household surveys was conducted in 1,461 adults from Curitiba, Brazil interviewed in person. The perceived environment was evaluated with the Neighborhood Environment Walkability Scale, and the POS use was evaluated using the ordinal scale (increased use).

**Results:**

The presence of interesting objects, heavy traffic, and the number of positive attributes of the environment was positively associated with POS use among men, and the presence of trees was associated with the use among women.

**Conclusions:**

Managers should invest in the architectural attractiveness of neighborhoods and should plant and conserve trees to encourage POS use.

## Introduction

Public open spaces (POS) such as parks, green areas and recreational facilities can help promote healthy living for people in urban areas by providing opportunities for physical activity practices and leisure activities. POS use can also promote physical and psychological well-being and facilitate the social integration of users [1-3]. Recent reviews have documented an association between proximity and access to POS with higher levels of physical activity in youths and adults [4,5]. However, these findings were reported in high-income countries such as North America, Europe and Oceania [1,2,6], which limits the applicability of the results to other social and economic contexts such as low- and middle-income countries.

In Latin American countries, there is little evidence that the presence and use of POS can provide benefits to public health. In Bogota, Colombia, the density of parks and the use of bike lanes are associated with better quality of life in adults [7]. In Curitiba, Brazil, the accessibility, presence and use of POS are associated with high levels of physical activity [8-10]. These results suggest that the availability of POS and easy community access can encourage greater participation in physical activity within a population.

Several characteristics of the physical and social environment may influence the choice and use of POS in high-income countries [1]. For example, the quality of the structures, access, maintenance, aesthetics and safety of a given area are positively associated with POS use [1,11]. Kaczynski et al. [12] found that the number of physical activity facilities is positively associated with physical activity practices in parks. Moreover, because most people bike or walk to their destinations in Brazil [10], it is possible that the characteristics of the neighborhood environment may favor the use of POS [1].

For example, the land use mix near the POS would stimulate residents to walk through the neighborhood to use shops and services in the neighborhood, which could facilitate park use. However, evidence shows an inverse association between the land use mix and park use [13]. In fact, the perception of aesthetics, street quality, traffic safety, crime and lighting of the neighborhood were associated with POS use among Australian and American adults [3,14,15]. However, there have been no similar studies in other countries. It is believed that people living in better quality neighborhoods would utilize the POS more frequently. The Brazilian population in Sao Paulo city represents one of the worst perceived indexes of safety and access to POS when compared with countries in North America, Europe, Asia and Oceania [16]. Another city in Brazil, Curitiba, is adopting strategies of urban planning that prioritize the construction and access to POS [17]. To date, there are 1,052 POS distributed in parks, plazas (small central parks), green areas and other places in Curitiba, which are constantly used by adults for physical activity [10,18].

According to a review conducted using several databases (Lilacs, SciELO, PubMed and Web of Science), the only study that examined the relationship between the perceived environment and POS use in countries with low and middle income was conducted by Reis et al. [19] and focused on adolescents from Curitiba. The authors found that decreased accessibility was inversely associated with physical activity practices in parks [19]. Additional information regarding this relationship in adults can help managers improve the neighborhood characteristics near a POS to increase its use by community members and benefit public health. The aim of this study was to investigate the association between the perceived neighborhood environment and POS use by adults in Curitiba, Brazil.

## Methods

### Design and place of study

In 2009, a cross-sectional household survey was conducted to evaluate the health characteristics, lifestyle, leisure habits and physical activity practices of adults living around parks and plazas in Curitiba.

Curitiba is the capital of Parana State (Southern Brazil) with a population of 1,746,896 inhabitants (100% urban, 52.3% women). It is the 8th largest city in the country, with a Human Development Index of 0.856 (high) [20], and to date, there are 17 green areas, 21 parks, 454 squares, 16 *“eixos de animação”* (recreational facilities) and 31 preservation areas dispersed in 75 neighborhoods. In addition, 29 sports and recreational centers offer organized activities for children, adolescents, adults and older adults. The recreational centers promote environmental preservation, and the city offers 64.5 m^2^ of green area per inhabitant, which is greater than the index of 15 m^2^/inhabitant recommended to promote good quality of life in Brazil [21]. The Municipal Secretary of Sports, Leisure and Youth and the Municipal Secretary of Health offer intervention programs for physical activity and the promotion of healthy habits in the population, which are conducted at recreational facilities [17].

### Public open space (POS) selection

Despite the high quantity of POS, some are not intended for physical activity practices. The locations were selected according to their potential for physical activity practices and were located in neighborhoods with different economic and environmental conditions to represent the characteristics of the adult population of the city. To select the study locations (POS), in the first phase, all 75 neighborhoods of the city were classified into nine strata based on a built and social environment (ENV) index for physical activity and income levels. The built environment information included park density (km^2^/inhabitants), plaza density (km^2^/inhabitants), bike lane density (km/inhabitants) and sports and leisure department units (# units/inhabitants) [20]. Crime rate (# crimes/inhabitants) and traffic accident data (# deaths/inhabitants) were used as social environmental indicators. Socioeconomic status (SES) was determined based on the average family income [17].

The ENV index was calculated by the sum of standardized built and social environment indicators to create a common scale (0–100) in which the product indicates the ENV score for each neighborhood. Higher values for this score indicate a better quality environment. The ENV index and SES in each neighborhood were categorized into tertiles and compared in a 3×3 matrix: high, medium, and low quality of ENV for physical activity practices and high, medium, and low SES. Using this matrix, the 75 neighborhoods were classified into nine strata.

The four neighborhoods located in the extreme clusters (high ENV and high SES, high ENV and low SES, low ENV and high SES, or low ENV and low SES) were selected to maximize environmental variability. The POS (parks and plazas) were selected using a mixed-methods approach. First, all the POS within each selected neighborhood were listed. The list was sent to coordinators from the Secretary of Health and Secretary of Sports and Leisure, who identified the locations in which interventions were delivered by their secretaries. After three rounds of consulting, four parks and four plazas were selected for evaluation through the consensus obtained in the consultation [22]. The evaluated plazas are considered to be “small parks” and differ from parks in total area, number and diversity of areas for physical activity. The details regarding the place selection and place characteristics are available in previous publications [17,22,23].

### Selection of residences

A 500-meter buffer was defined around each of the eight POS, and all the streets within this buffer were visited to identify street segments that were residential, commercial or mixed (n = 1,899). Twenty-nine percent of the street segments were not residential and were excluded from the study (n = 361). The residential segments previously identified were ordered using ArcGis software. Finally, to establish geographic representation, one residence was randomly selected in each of the 1,538 eligible segments.

### Participant selection

Eligible participants were defined as adults (≥18 yr) living in a given neighborhood for at least one year. All the selected households were visited in person, and the number of eligible residents was listed at the time of the visit. Study participants were randomly selected from the total eligible residents of the house [24]. A total of three attempts were made on different days and times to contact the subject. Individuals who did not live in the household (e.g., maids and visitors), individuals with physical impairments that limit physical activity practice and individuals with cognitive limitations in understanding the questions were excluded.

### Number of participants and data collection

Face-to-face interviews were conducted in 95% of the eligible segments, which resulted in a total sample of 1,461 participants. In the statistical analysis, a posteriori [25] showed that the sample (530 men and 931 women) had a power of ≥82% (β = 20%) and a confidence level of 95% (α = 5%) to detect odds ratio (OR), which was identified as significant in this study.

The overall refusal rate was 7.9% (n = 121). Quality control of the data collection was performed by field supervisors who reinterviewed 74 participants (12.5% of the sample). Eight trained interviewers, all women with high school degrees, conducted the interviews after completing 30 training hours. Data collection occurred between April and July of 2009. The study was approved by the Internal Review Board at the Federal University of Pelotas (005/2008) and written informed consent was obtained from the participants for publication of this report.

### Outcome variable

The use of a specific POS near a residence was determined by asking the following question: “In the last 12 months, did you use the park/plaza?” (yes/no). The frequency of POS use was quantified based on a question with nine response options (“a few times a year”, “a few times a month” and “one” to “seven times per wk”).

For analytical purposes, POS use was classified on an ordinal scale with four levels: “no use” (0), “use a few times a year/month” (1), “use between 1–3 times/wk” (2) and “use ≥4 times/wk” (3). The test-retest reliability of the POS use measurement was adequate (82 – 92% agreement, kappa 0.65 – 0.77, p < 0.001).

### Independent variables

The perceived environment was assessed with a modified and culturally adapted version of the Neighborhood Environment Walkability Scale (NEWS) [26] with dichotomous response options (yes/no). The scale was translated, adapted and validated for the Brazilian context [27] and is frequently used in research concerning the association between the perceived environment and physical activity practices [9,28,29]. The pilot study and previous research conducted in Brazil showed that people have difficulty understanding questions when response options are presented on a Likert scale [30].

Based on the literature review [1,3,14], twelve questions about perceptions of neighborhood characteristics that are potentially associated with POS use were selected and divided into three categories with four items each: (i) aesthetics, (ii) traffic safety and (iii) crimes safety. The study participants reported the perception of (i) the presence of trees (“*There are trees along the streets in my neighborhood”*), attractiveness (*“There are many interesting things to look at while walking in my neighborhood”*), beautiful landscapes and buildings in the neighborhood (*“There are many attractive natural sights in my neighborhood”* and *“There are attractive buildings/homes in my neighborhood”*); (ii) traffic conditions such as flow and speed of vehicles (*“There is so much traffic along nearby streets that it makes it difficult or unpleasant to walk in my neighborhood”* and *“The speed of traffic on most nearby streets is usually slow”*), disregard for speed limits (“*Most drivers exceed the posted speed limits while driving in my neighborhood”*) and the presence of facilities for crossing streets (*“There are crosswalks and pedestrian signals to help walkers cross busy streets in my neighborhood”);* and (iii) the presence of people walking/cycling *(“Walkers and bikers on the streets in my neighborhood can be easily seen by people in their homes”)*, crime (*“There is a high crime rate in my neighborhood”)* and safety for walking during the day *(“The crime rate in my neighborhood makes it unsafe to go on walks during the day”)* and night *(“The crime rate in my neighborhood makes it unsafe to go on walks at night”)* through the streets of the neighborhood. The test-retest reliability of these variables was adequate in a subsample of participants (70.2 – 88.6% agreement, kappa 0.39 – 0.68, p < 0.05).

The association between each environmental variable and park/plaza use was tested. Additionally, based on the evidence that a combination of perceived environmental attributes are associated with physical activity in different countries [16], a variable was created by the sum of the 12 available items to represent the number of positive attributes within each neighborhood (environmental score). For this evaluation, the variables “traffic”, “drivers exceed the speed limit” and “crime in the neighborhood” were recoded (from 0 to 1). The absence of any of these attributes indicates a positive aspect of the environment. The score of the variable “number of positive attributes of the environment” varied between 0 and 12 points (indicating low and high quality, respectively) and was subsequently classified into three categories: 0–5 items (lower quality), 6–7 items and ≥ 8 items (higher quality).

### Covariates

Participants were grouped into two age groups (18–39.9 and ≥40 years). The methodology proposed by *“Associação Brasileira das Empresas de Pesquisa”*[31] was used to determine SES based on the number of appliances within the household (e.g., television, washing machine), the presence of maids and education level. Based on their household score, the participants were classified into two levels: “low” and “high”. The perceived health was evaluated on a four-point Likert-type scale (“poor”, “regular”, “good” and “very good”) with the following question: “How do you consider your health?” [32]. The response options “good” and “very good” were combined to characterize the variable “positive perceived health”.

### Data analysis

The chi-squared (χ^2^) test for linear trends and heterogeneity was used to compare the proportions of POS use among the categories of independent variables (each environment variable separately and the environment score). Ordinal logistic regression with proportional odds model [33] was used to analyze the association between the perceived environment and POS use with the ordinal outcome categorized into four levels: “no use = 0; “use a few times a year/month = 1”; “use between 1–3 times/wk = 2” and “use ≥4 times/wk = 3”. Initially, a variance inflation factor test (VIF) was performed, which rejected the hypothesis of multicollinearity (1/VIF ≥ 0.62). All the variables were considered for inclusion in the multiple regression model; however, only the variables with p < 0.20 remained in the final model. The variables were inserted at the same level of analysis using the method of forced entry. The following confounding factors were considered: age, SES and perceived health. Under the premise of proportional odds, the Brant test confirmed the explanatory variables individually for men and women with p values ≥0.18 and 0.38, respectively. Analyses were performed using Stata 11, and the procedure adopted in the selection of the sample into consideration with the command “svy” was used to correct the estimates of variability of POS use. Because the analysis was designed to verify a significant interaction between sex and POS use, study participants were stratified by sex, and the level of significance was maintained at 5%.

## Results

Most participants were ≥ 40 years old (58.5%) with high SES (62.1%) and positive perceived health (71.3%). There was a difference between sexes (p < 0.05) where men presented higher SES and perceived health (Table 1). The perception of safety while walking in the neighborhood was higher in men (p < 0.05). Approximately eight out of ten participants reported that it is safe to walk during the day, but the same proportion of people felt unsafe walking at night. The proportion of participants who perceived eight or more positive items in the neighborhood environment was higher among men (p = 0.004). Approximately six out of ten participants reported using the POS near the residence, and approximately one out of ten participants reported using the POS at least four times a week. The proportions of men and women in each category of parks/plazas were similar (p = 0.101).

**Table 1:** Sociodemographic characteristics, perceived environment and public open space use by adults from Curitiba, Brazil, 2009 (n = 1,461)

	**All (n = 1,461)**		**Men (n = 530)**		**Women (n = 931)**		**p**
	**n**	**%**	**n**	**%**	**n**	**%**	
Age (yrs)							
18–39.9	604	41.5	238	44.9	366	39.5	**0.045**^**a**^
≥ 40	852	58.5	292	55.1	560	60.5	
Economic level							
Low	551	37.9	174	33.0	377	40.7	**0.004**^**a**^
High	902	62.1	353	67.0	549	59.3	
Perceived health							
Negative	420	28.7	126	23.8	294	31.6	**0.002**^a^
Positive	1041	71.3	404	76.2	637	68.4	
**Neighborhood aesthetics**							
Trees in the streets							
No	312	21.4	103	19.5	209	22.5	0.179^a^
Yes	1147	78.6	426	80.5	721	77.5	
Interesting things to see							
No	757	51.9	263	49.7	494	53.1	0.211^a^
Yes	702	48.1	266	50.3	436	46.9	
Natural attractions							
No	756	51.8	264	49.8	492	52.9	0.256^a^
Yes	704	48.2	266	50.2	438	47.1	
Beautiful buildings and houses							
No	357	24.5	131	24.8	226	24.3	0.843^a^
Yes	1102	75.5	398	75.2	704	75.7	
**Traffic safety**							
Heavy traffic makes it difficult to walk							
No	552	37.8	200	37.7	352	37.8	0.966^a^
Yes	908	62.2	330	62.3	578	62.2	
Low traffic speed							
No	685	46.9	240	45.3	445	47.8	0.345^a^
Yes	775	53.1	290	54.7	485	52.2	
Drivers exceed the speed limit							
No	991	67.9	357	67.4	634	68.2	0.749^a^
Yes	469	32.1	173	32.6	296	31.8	
Tracks and signs facilitate the crossing of streets							
No	901	61.7	331	62.5	570	61.3	0.660^a^
Yes	559	38.3	199	37.5	360	38.7	
**Crimes safety**							
People walking in the street							
No	596	40.8	199	37.6	397	42.7	0.058^a^
Yes	863	59.2	330	62.4	533	57.3	
Crimes in the neighborhood							
No	779	53.4	277	52.3	502	54.0	0.528^a^
Yes	681	46.6	253	47.7	428	46.0	
Safe to walking during the day							
No	243	16.7	68	12.8	175	18.8	**0.003**^**a**^
Yes	1216	83.3	462	87.2	754	81.2	
Safe to walking during the night							
No	1143	78.3	381	71.9	762	81.9	**<0.001**^**a**^
Yes	317	21.7	149	28.1	168	18.1	
Number of positive attributes of the environment							
0–5 items	411	28.2	125	23.8	286	30.8	
6-7 items	560	38.5	208	39.5	352	37.9	**0.004**^**b**^
≥8 items (higher quality)	484	33.3	193	36.7	291	31.3	
Public open space use							
No use	572	39.2	186	35.1	386	41.5	**0.101**^**b**^
Use a few times a year/month	419	28.7	165	31.1	254	27.3	
Use between 1–3 times/wk	307	21.0	119	22.5	188	20.2	
Use ≥4 times/wk	163	11.2	60	11.3	103	11.1	

a: χ^2^ to heterogeneity; b: χ^2^ to linear trend.

In the bivariate analysis for men (Table 2), the frequency of POS use was higher among participants who reported the presence of interesting things, natural attractions, safety of walking during the night and a higher number of positive attributes of the environment (p < 0.05). Among women, the frequency of POS use was higher among those who reported the presence of trees, interesting things to see, natural attractions, beautiful buildings/homes, disrespect for speed limits, crime, more safety for walking during the day and a higher number of positive attributes of the environment (p < 0.05).

**Table 2:** Distribution of public open space use frequency according with the characteristics of the perceived environment by adults from Curitiba, Brazil, 2009 (n = 1,461)

	**Men (n = 530)**				**Women (n = 931)**			
	**No use**	**Use a few times a year/month**	**Use between 1–3 times/wk**	**Use ≥4 times/wk**	**No use**	**Use a few times a year/month**	**Use between 1–3 times/wk**	**Use ≥4 times/wk**
	**%**	**%**	**%**	**%**	**%**	**%**	**%**	**%**
Trees in the streets								
No	39.8	34.9	17.5	7.8	49.8	29.2	12.9	8.1
Yes	33.8	30.3	23.7	12.2	39.1	26.8	22.2	11.9^a^
Interesting things to see								
No	42.6	32.3	17.5	7.6	45.8	26.9	17.6	9.7
Yes	27.8	30.1	27.1	15.0^b^	36.7	27.8	22.9	12.6^a^
Natural attractions								
No	44.3	29.6	16.3	9.8	47.2	26.4	16.9	9.6
Yes	25.9	32.7	28.6	12.8^b^	35.2	28.3	23.7	12.8**
Beautiful buildings and houses								
No	40.5	28.2	20.6	10.7	48.7	24.8	17.3	9.3
Yes	33.4	32.2	22.9	11.6	39.2	28.1	21.0	11.7^a^
Heavy traffic makes it difficult to walk								
No	42.5	27.5	18.5	11.5	43.2	28.4	19.0	9.4
Yes	30.6	33.3	24.9	11.2	40.5	26.6	20.8	12.1
Low traffic speed								
No	37.9	30.4	21.3	10.4	42.0	27.2	17.5	13.3
Yes	32.8	31.7	23.4	12.1	41.0	27.4	22.5	9.1
Drivers exceed the speed limit								
No	37.8	29.1	23.0	10.1	43.5	27.6	18.0	10.9
Yes	29.5	35.3	21.4	13.9	37.2	26.7	24.7	11.5^a^
Tracks and signs facilitate the crossing of streets								
No	35.0	31.7	21.8	11.5	43.3	27.4	19.7	9.6
Yes	35.2	30.2	23.6	11.1	38.6	27.2	20.8	13.3
People walking in the street								
No	37.2	33.7	20.6	8.5	42.8	25.7	20.7	10.8
Yes	33.9	29.7	23.3	13.0	40.5	28.5	19.7	11.3
Crimes in the neighborhood								
No	38.3	30.0	19.9	11.9	45.2	26.5	18.7	9.6
Yes	31.6	32.4	25.3	10.7	37.1	28.3	21.7	12.9^a^
Safe to walking during the day								
No	41.2	27.9	23.5	7.4	52.0	24.6	13.7	9.7
Yes	34.2	31.6	22.3	11.9	39.0	28.0	21.6	11.4^a^
Safe to walking during the night								
No	38.1	30.7	20.2	11.0	42.4	26.9	20.2	10.5
Yes	27.5	32.2	28.2	12.1^a^	37.5	29.2	19.6	13.7
Number of positive attributes of the environment								
0–5 items	44.8	32.0	15.2	8.0	49.0	23.4	17.5	10.1
6–7 items	36.5	33.7	19.7	10.1	40.3	30.4	18.8	10.5
≥8 items (higher quality)	27.5	28.5	29.0	15.0^b^	35.4	27.5	24.4	12.7^a^

^a^p < 0.05 and ^b^p < 0.001 on χ^2^ test for linear trend.

In the bivariate ordinal logistic regression (Table 3), positive associations were found between the perception of interesting things in the neighborhood (OR: 2.02, CI_95%_: 1.53-2.68), the crime rate (OR: 1.33, CI_95%_: 1.01-1.75) and the number of positive attributes of the environment (OR: 2.32, CI_95%_: 1.21-4.43) with POS use by men. For women, only the presence of trees was associated with the use of POS (OR: 1.62, CI_95%_: 1.03-2.56).

**Table 3:** Ordinal logistic regression for the association between perceived environment and public open space use* by adults from Curitiba, Brazil, 2009 (n = 1,461)

	**Men (n = 530)**				**Women (n = 931)**			
	**Bivariate analysis**		**Multivariate analysis**^**†**^		**Bivariate analysis**		**Multivariate analysis**^**†**^	
	**OR**	**CI**_**95%**_	**OR**	**CI**_**95%**_	**OR**	**CI**_**95%**_	**OR**	**CI**_**95%**_
Trees in the streets								
No	1		1		1		1	
Yes	1.47	0.86-1.25	1.15	0.72-1.84	**1.62**	**1.03-2.56**	**1.58**	**1.04-2.39**
Interesting things to see								
No	1		1		1		1	
Yes	**2.02**	**1.53-2.68**	**2.07**	**1.57-2.73**	1.43	0.95-2.14	1.28	0.92-1.79
Natural attractions								
No	1		1		1		1	
Yes	1.99	0.95-4.16	1.56	0.64-3.78	1.58	0.86-2.89	1.44	0.82-2.55
Beautiful buildings and houses								
No	1		1		1		1	
Yes	1.24	0.63-2.41	0.93	0.52-1.67	1.42	0.70-2.87	1.22	0.65-2.28
Heavy traffic makes it difficult to walk								
No	1		1		1		1	
Yes	1.42	0.93-2.19	**1.58**	**1.04-2.40**	1.16	0.70-1.91	1.08	0.65-1.80
Low traffic speed								
No	1		1		1		1	
Yes	1.21	0.68-2.15	1.03	0.62-1.73	0.99	0.76-1.30	0.95	0.71-1.28
Drivers exceed the speed limit								
No	1		1		1		1	
Yes	1.27	0.90-1.78	1.10	0.78-1.56	1.30	0.92-1.83	1.22	0.88-1.70
Tracks and signs facilitate the crossing of streets								
No	1		1		1		1	
Yes	1.01	0.71-1.41	0.91	0.69-1.20	1.24	0.77-1.99	1.15	0.73-1.81
People walking in the street								
No	1		1		1		1	
Yes	1.28	0.82-1.99	1.24	0.85-1.79	1.04	0.81-1.34	0.98	0.74-1.30
Crimes in the neighborhood								
No	1		1		1		1	
Yes	**1.33**	**1.01-1.75**	1.34	0.95-1.89	1.32	0.82-2.14	1.17	0.73-1.89
Safe to walking during the day								
No	1		1		1		1	
Yes	1.29	0.86-1.94	1.28	0.96-1.70	1.57	0.99-2.48	1.41	0.89-2.22
Safe to walking during the night								
No	1		1		1		1	
Yes	1.49	0.74-2.99	1.36	0.65-2.84	1.26	0.83-1.90	1.05	0.73-1.49
Number of positive attributes of the environment								
0–5 items	1		1		1		1	
6–7 items	1.39	0.97-2.00	1.35	0.98-1.87	1.27	0.73-2.22	1.26	0.78-2.04
≥8 items (higher quality)	**2.32**	**1.21-4.43**	**2.31**	**1.27-4.20**	1.64	0.97-2.79	1.56	0.97-2.52

*variable classified in four categories: no use = 0; use a few times a year/month = 1; use between 1–3 times/wk = 2 and use ≥4 times/wk = 3. † Adjusted for variables with p < 0.20 in initial multivariate analysis.

In the multiple regression analyses (Table 3), the presence of interesting things to see in the neighborhood and the number of positive environmental attributes remained associated with the frequency of POS use by men. The perception of heavy traffic gained significance in the multivariate analysis, while the perception of crime decreased in the magnitude of the measure of association. Among women, the presence of trees remained associated with the frequency of POS use (OR: 1.58, CI_95%_: 1.04-2.39).

**Figure 1:**
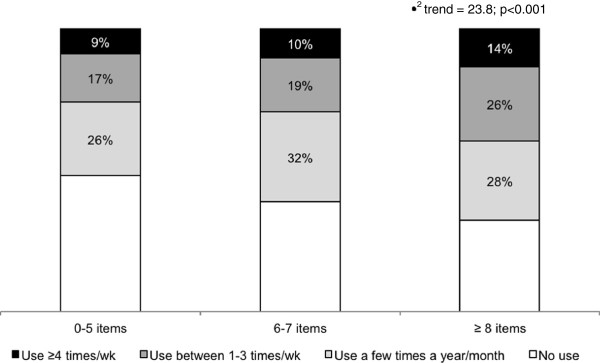
Association between number of positive attributes of the environment and public open space use by adults from Curitiba, Brazil, 2009 (n=1,461).

Another important result of this study was to identify a linear association between the number of positive attributes of the environment and the frequency of POS use (p < 0.001) (Figure 1); results were maintained when the data were stratified by sex (data not shown).

## Discussion

This is one of the first studies to explore the association between the perceived environment and POS use in adults from Latin America. The methodology used is an important strength of the research, and it allowed the representation of environmental and social neighborhood attributes. The selection of neighborhoods allowed the inclusion of parks and plazas from areas with different income and built environment characteristics. Additionally the selection of parks and plazas followed a comprehensive approach allowing the identification of places that were more likely to be used by the population as indicated by the secretariats coordinators.

Significant differences were found between men and women. For men, the perception of interesting things, heavy traffic and the number of positive attributes of the environment were associated with frequent POS use. For women, only the presence of trees in the neighborhood streets increased the likelihood of using the spaces. These findings indicate that favorable environments can stimulate POS use. The results should guide interventions and policies to increase the use of POS in similar contexts.

Evidence shows that the perceived environmental attributes of neighborhoods such as aesthetics, safety from crime and traffic are associated with the practice of walking for leisure [9,15,34]. This finding is relevant because 70% of people walk in neighborhood streets [35]. In Pelotas (South Brazil), 73% of adults walk at least one day a week and 41% walk more than 150 min/wk, and walking for leisure is the most frequent physical activity practiced by adults [36,37]. Leslie et al. [3] found a positive association between park use and walking for leisure among Australian adults. Additionally, Koohsari et al. [15] found that the perception of neighborhood aesthetics increased the odds of adults walking to and in the neighborhood parks by 117%. In the present study, the perception of neighborhood attractiveness increased the odds of POS use among men. This association may be explained by the scenery observed during the commute to a POS, which encourages the practice of walking in neighborhood streets [38] and in the POS near the home. Eyler et al. [35] found that 36% of people walk in parks regularly. The neighborhood aesthetics were potentially associated with the practice of walking among men but not among women [38]. Leslie et al. [3] found no association between perceived aesthetics and monthly use of the parks; however, their analysis was not stratified by sex as in the present study.

Men who perceived traffic were more likely to use POS. However, others studies found a positive association between perceptions of low vehicle speed, light traffic volume, higher safety from traffic and the use of POS such as parks and bike lanes [3,14,15]. The association found in the present study can be attributed to men being more physically active in commuting to the POS. Men are more likely to be exposed to heavy traffic in the neighborhood and on city streets because Curitiba has the highest ratio of vehicles per capita in the country (1:2) [9]. Despite the lack of association between the traffic safety perception and the practice of walking for leisure in Curitiba adults [9], excess vehicles and heavy traffic can pollute the streets and make walking unpleasant. Brownson et al. [39] found a positive association between the perception of traffic and physical activity. It is possible that the proximity, the green areas and the trails available in the POS favor its use [11,22,34,38]. In fact, the POS use in Curitiba is associated with walking for leisure [10], and the activity was reported more frequently by respondents (52%, data not shown).

For women, the presence of trees in the neighborhood street increased the likelihood of POS use. Curitiba has one of the highest levels of green area per inhabitant in Brazil (64.5 m^2^/inhabitant), and most of these areas are located in parks, plazas and green areas within the city. We believe that the home proximity to POS may have favored the observed association [11]. The physical characteristics of parks and the presence of green areas can attract individuals, especially women, to leisure activities [1,11]. The association for women alone can be explained by the characteristics of the occupation of the POS because many women use green areas for walking [2]. In Brazil, walking is a common physical activity for leisure among women (61%) [37], and it occurs at a high frequency in parks and plazas in Curitiba. Among POS users, 57% of women and 44% of men reported the use of the POS for walking (data not shown).

The association between the number of positive attributes of the environment and POS use (Figure 1) reinforces the idea that the full set of attributes of a neighborhood (aesthetics, traffic and crimes safety) encourages park use rather than any feature alone. The lack of association among women (Table 3) suggests an interaction effect between the variables. In the analysis of all the participants, the highest environmental quality (≥8 items) was associated with POS use (OR: 1.81, CI_95%_: 1.11-2.93). These results can be partly explained by inequalities related to leisure opportunities between sexes. In Brazil, women spend three times more time performing house work than men [40]. This accumulation of tasks can reduce the availability of time for leisure and negatively influence the perception of opportunities for the use of parks independently of the quality of the environment. In general, POS use was higher among men (65% *vs.* 59%, p = 0.018).

### Limitations

Some limitations should be considered when interpreting the results. The sample was not representative of the city because it was restricted to adults living around the POS with potential for physical activity. Thus, the results cannot be extrapolated to the whole community. A high quantity of POS in Curitiba is responsible for 77% of the population reporting the existence of places close to the home for physical activity practices [41]. This characteristic may explain the lack of association between most of the environmental attributes and POS use. Since 1998, the city council invested in an intervention program to promote physical activity (*CuritibAtiva*) with structured activities for the community [17]. Much of the program’s actions occur in parks, plazas, and other POS, which can stimulate POS use independently of the environmental quality of the neighborhood. Additionally, the quality of features present within the selected POS (e.g., green areas, recreational facilities and tree coverage) was not analyzed. Therefore, the study did not account for the relative importance of such attributes in POS use. While the study provides data on POS use, it does not indicate how the parks and plazas were used. It also does not provide data on the physical activities (frequency, intensity, type or time) performed within the POS.

## Conclusions

The presence of attractions, heavy traffic, trees and the number of positive attributes of the neighborhood environment were all associated with increased POS use. Managers should invest in improving and maintaining these environmental characteristics in neighborhoods close to parks to facilitate and/or encourage POS use. Investing in architectural projects to improve the aesthetics of buildings and public works and planting and conservation of trees and green areas should be encouraged to make the neighborhood more pleasant and attractive and increase POS use. The use of POS can promote physical activity and, consequently, the physical and psychological well-being of a given population. Because the quality of facilities for physical activity practices are important factors to POS use, future studies should consider the role of the features, conditions, accessibility, aesthetics, safety and social environment in the POS use patterns in low- and medium-income countries.

## Competing interests

The authors declare that they have no conflict of interests.

## Authors’ contributions

RC Fermino and RS Reis conceived and designed the study, prepared the data, conducted the analysis and data interpretation, and wrote the manuscript. PC Hallal contributed to the study design and reviewed the manuscript for important intellectual content. JC Farias Júnior reviewed the manuscript for important intellectual content. All the authors read and approved the final version.
